# Pulmonary perfusion by chest digital dynamic radiography: Comparison between breath‐holding and deep‐breathing acquisition

**DOI:** 10.1002/acm2.13071

**Published:** 2020-10-26

**Authors:** Shota Yamamoto, Terumitsu Hasebe, Kosuke Tomita, Shunsuke Kamei, Tomohiro Matsumoto, Yutaka Imai, Genki Takahashi, Yusuke Kondo, Yoko Ito, Fumio Sakamaki

**Affiliations:** ^1^ Department of Radiology Tokai University Hachioji Hospital Tokai University School of Medicine Hachioji Tokyo Japan; ^2^ Department of Respiratory Medicine Tokai University Hachioji Hospital Tokai University School of Medicine Hachioji Tokyo Japan

**Keywords:** digital dynamic radiography, pulmonary perfusion, respiratory disease, respiratory artifact, x‐ray

## Abstract

**Purpose:**

Pulmonary perfusion is an important factor for gas exchange. Chest digital dynamic radiography (DDR) by the deep‐breathing protocol can evaluate pulmonary perfusion in healthy subjects. However, respiratory artifacts may affect DDR in patients with respiratory diseases. We examined the feasibility of a breath‐holding protocol and compared it with the deep‐breathing protocol to reduce respiratory artifacts.

**Materials and methods:**

A total of 42 consecutive patients with respiratory diseases (32 males; age, 68.6 ± 12.3 yr), including 21 patients with chronic obstructive pulmonary disease, underwent chest DDR through the breath‐holding protocol and the deep‐breathing protocol. Imaging success rate and exposure to radiation were compared. The correlation rate of temporal changes in each pixel value between the lung fields and left cardiac ventricles was analyzed.

**Results:**

Imaging success rate was higher with the breath‐holding protocol vs the deep‐breathing protocol (97% vs 69%, respectively; *P* < 0.0001). The entrance surface dose was lower with the breath‐holding protocol (1.09 ± 0.20 vs 1.81 ± 0.08 mGy, respectively; *P* < 0.0001). The correlation rate was higher with the breath‐holding protocol (right lung field, 41.7 ± 9.3%; left lung field, 44.2 ± 8.9% vs right lung field, 33.4 ± 6.6%; left lung field, 36.0 ± 7.1%, respectively; both lung fields, *P* < 0.0001). In the lower lung fields, the correlation rate was markedly different (right, 15.3% difference; left, 14.1% difference; both lung fields, *P* < 0.0001).

**Conclusion:**

The breath‐holding protocol resulted in high imaging success rate among patients with respiratory diseases, yielding vivid images of pulmonary perfusion.

## 1 INTRODUCTION

Pulmonary perfusion is the circulation of blood from the right ventricle to the left atrium and involved in gas exchange.^1^ Therefore, the evaluation of pulmonary circulation is of vital importance for the management of numerous respiratory diseases. Pulmonary perfusion is generally evaluated based on examinations using contrast media or radioactive agents.^2^, ^3^ Computed tomography (CT) and magnetic resonance imaging (MRI) have been beneficial in assessing the physiological function of the lungs.^4^, ^5^ However, CT and MRI have disadvantages, including high doses of radiation in CT scanning,^6^ long duration of examination, and substantial financial burden associated with MRI.^7^ In these diagnostic imaging methods, the patients need to be in the supine position, and imaging in the upright position is challenging.

Chest digital dynamic radiography (DDR) has been developed for the novel evaluation of pulmonary perfusion.^8^, ^9^, ^10^ Digital dynamic radiography technology rapidly captures sequential radiographs in a single examination to observe the dynamic interactions of anatomical structures. The lungs always hold approximately 500 ml of blood, and approximately 75 ml of blood is transferred into the vessels during a single cardiac cycle. The pulsatile blood flow is captured on the image as a change in the pixel value of the pulmonary vessels. Technically speaking, the pixel value increases very slightly during systole and decreases very slightly during diastole. Chest DDR can detect the change in pixel value synchronized with the cardiac cycle, and generate vivid pulmonary perfusion images.^8^, ^11^, ^12^ Its duration is approximately 15 s, it does not require contrast media, and can be performed in the standing, seated, and supine positions. Owing to the high sensitivity of the flat panel detector, the total exposure to radiation can be less than the limits for two projections recommended by the International Atomic Energy Agency (1.9 mGy).^13^ Based on these characteristics, chest DDR may be beneficial to screening for pulmonary embolism or pulmonary hypertension.^8^ A previous animal‐based study of a porcine pulmonary embolism model has revealed that chest DDR was able to detect perfusion defects in the lobe unit.^14^ In clinical practice, it has been reported that chest DDR could detect pulmonary perfusion defects in patients with giant cell arteritis and chronic thromboembolic pulmonary hypertension.^15^, ^16^ Moreover, DDR images were remarkably similar to those of pulmonary angiography, the iodine mapping images acquired with dual‐energy CT, and those of lung perfusion scintigraphy.^11^, ^12^

The deep‐breathing protocol is an imaging protocol that has been used since the dawn of DDR technology.^8^ It was an innovative method for the simultaneous evaluation of both ventilation and pulmonary blood flow by pausing for 2 s between deep breaths. However, respiratory artifacts are an important challenge for the examination of patients with respiratory diseases. Incomplete diaphragm pause in chest DDR could lead to two types of respiratory artifacts, namely blurred images and misalignment of the rib cage. In a previous study of chest DDR conducted by Tanaka et al., breath‐holding at rest expiratory level was helpful in capturing vivid pulmonary perfusion images.^12^ Nevertheless, the appropriate duration of breath‐holding or levels (e.g., maximum or rest, expiratory or inspiratory) are unclear. For clinical use, a shorter breath‐holding time is preferable. Thus, we sought to investigate whether breath‐holding for 6 s (breath‐holding protocol) at the maximum inspiratory level could reduce respiratory artifacts in clinical practice.

For this purpose, we examined the feasibility of the breath‐holding protocol and compared it with that of the deep‐breathing protocol, as previously reported.^8^

## 2 MATERIALS AND METHODS

### 2.A Patients

**Fig. 1:**
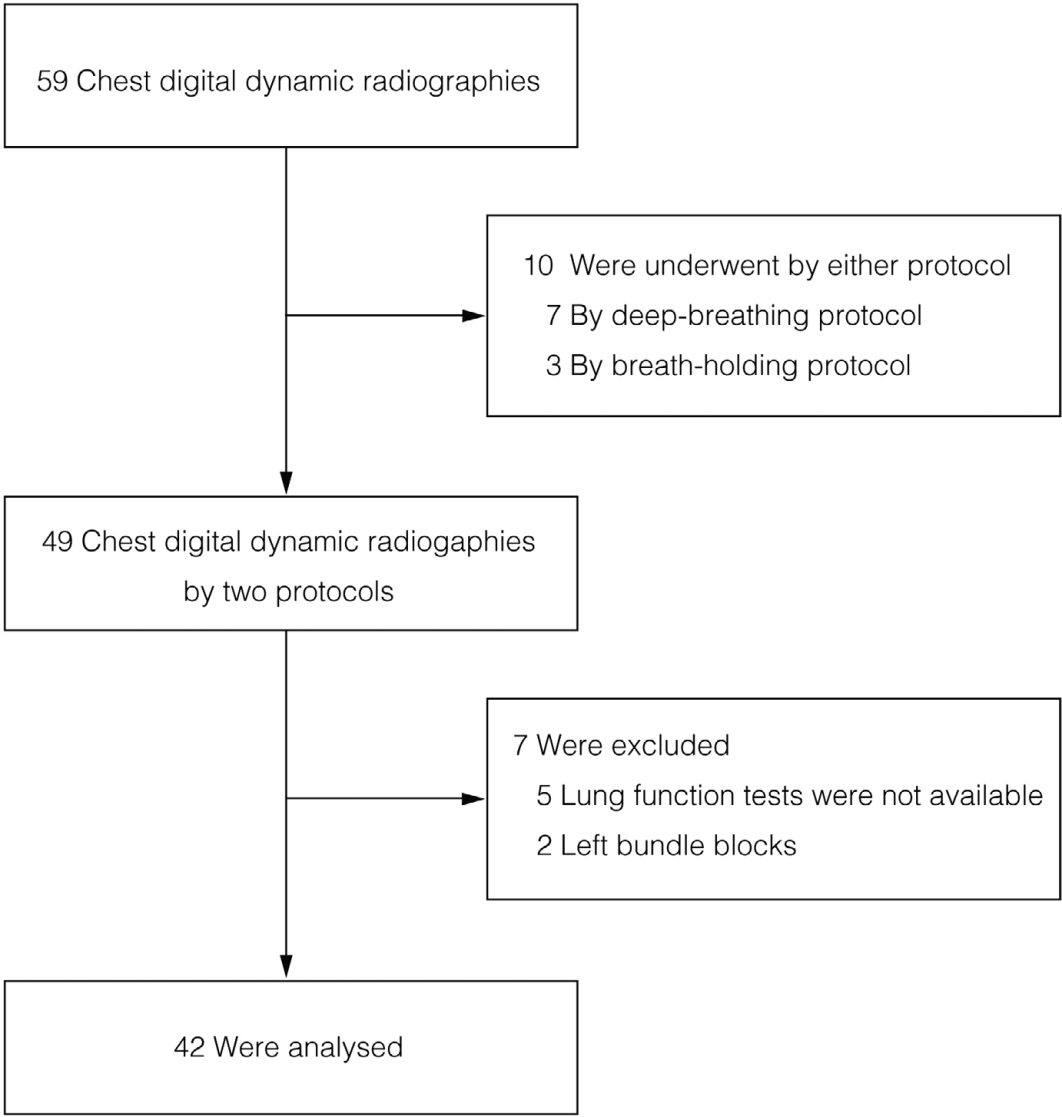
The flowchart of patient selection.

From February 2019 to January 2020, 44 consecutive patients with respiratory diseases who visited our hospital were investigated. The inclusion criteria for patients were: age >20 yr; chest DDR by both protocols; and a lung function test. Initially, 59 patients were photographed during the relevant period. The two protocols were utilized in 49 patients. After excluding five patients who did not undergo a lung function test and two patients with left bundle block, 42 patients were included in the analysis (Fig. 1). Given the principle of imaging, the reliability of pulmonary perfusion images could be reduced in patients with left bundle block. Heights and weights of the patients were measured to calculate the body mass index. Heart rate, smoking status, as well as details on respiratory and cardiovascular diseases were collected from the medical records of the patients.

### 2.B Study design and compliance with ethical guidelines

This was a retrospective case–control study investigating the feasibility of the breath‐holding protocol in chest DDR. The research was conducted according to the principles of the Declaration of Helsinki. Written informed consent was provided by all patients prior to their participation. This study has been reviewed based on the Clinical Trials Act and approved as a specified clinical trial.

### 2.C Imaging protocol of chest DDR

**Fig. 2:**
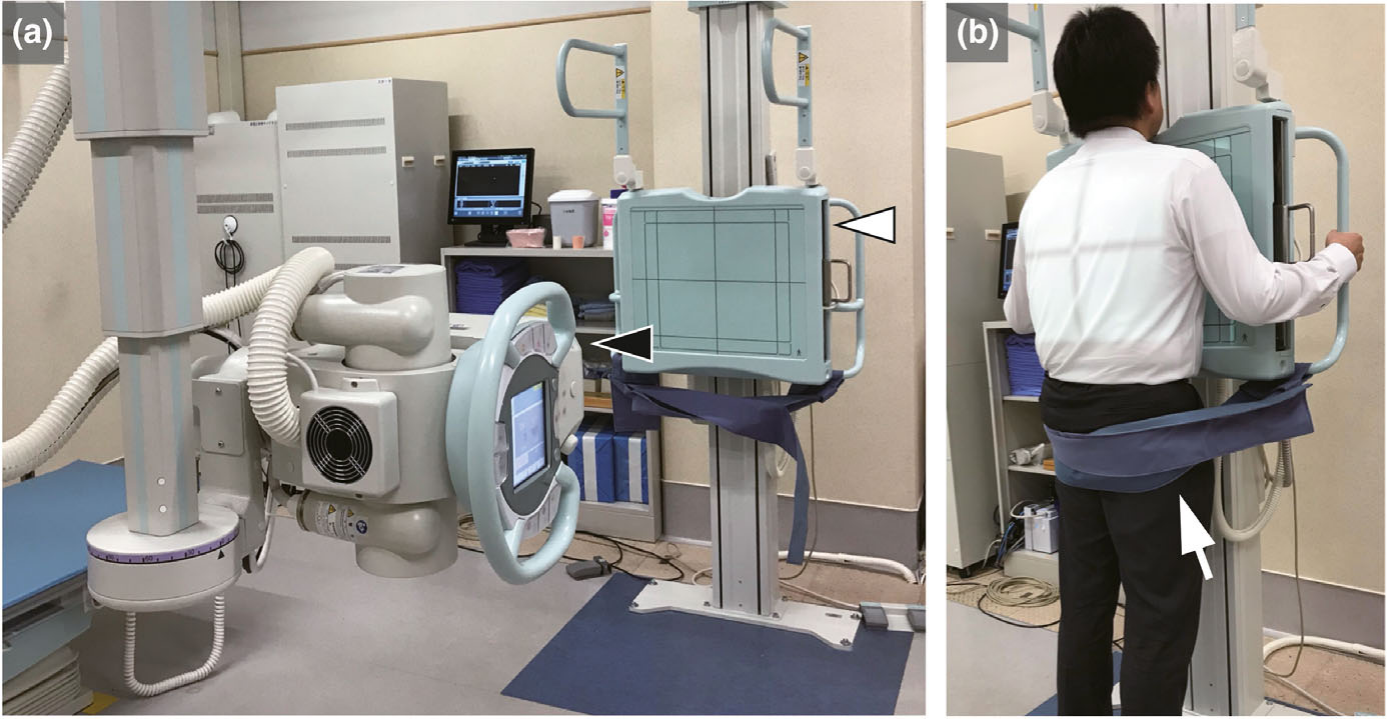
Clinical practice of chest digital dynamic radiography (DDR). (a) Flat panel detector system (Konica Minolta, Inc., Tokyo, Japan) was composed of a flat panel detector (AeroDR fine; Konica Minolta, Inc., Tokyo, Japan) (white arrowhead) and a pulsed x‐ray generator (RAD speed Pro; Shimadzu Corporation, Kyoto, Japan) (black arrowhead). In addition to chest DDR, conventional chest radiographs can also be taken. (b) Standing posteroanterior position in chest digital dynamic radiography. In order to minimize motion artifact, the pelvis is fastened firmly by a belt (white arrow).

**Fig. 3:**
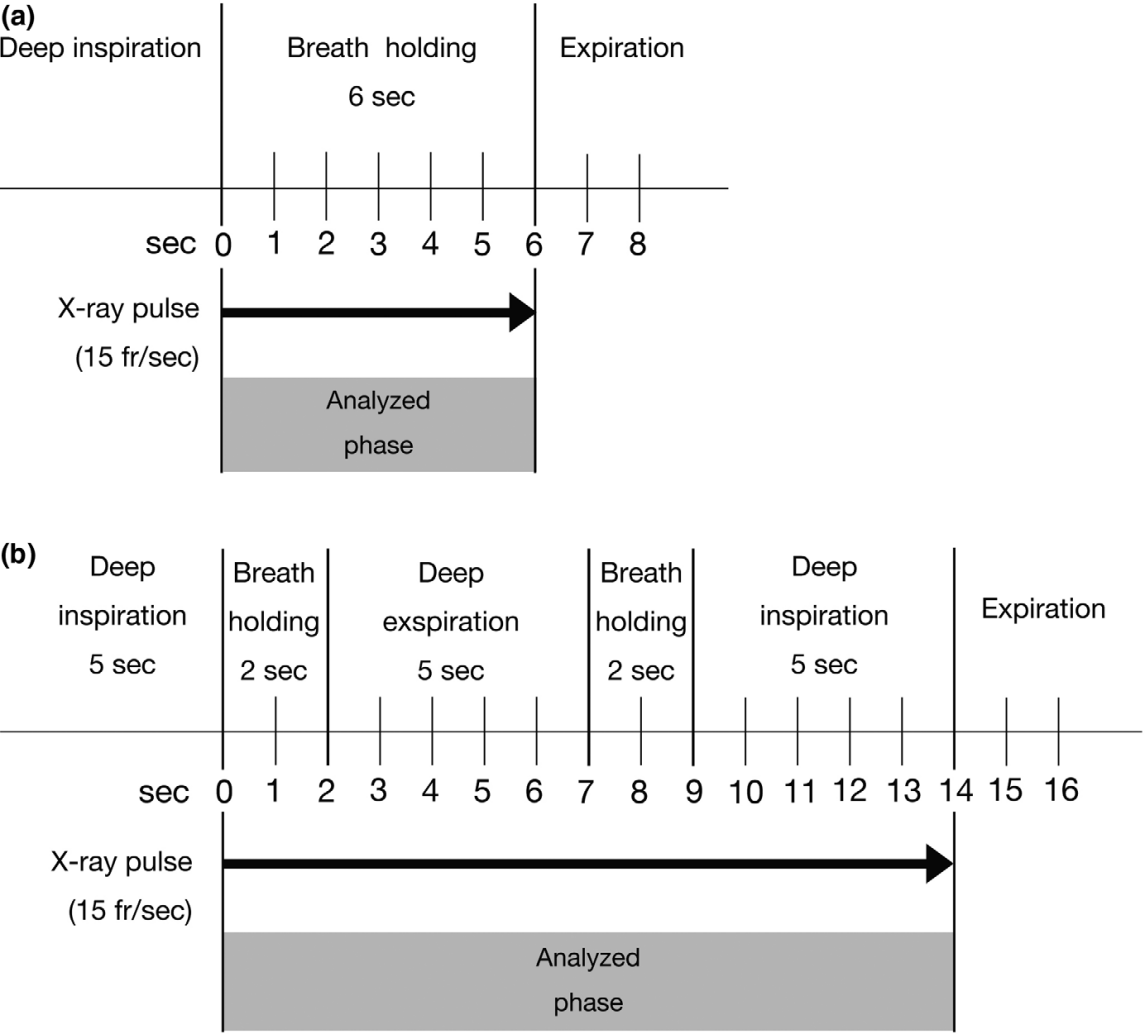
The imaging protocol: (a) Breath‐holding protocol. (b) Deep‐breathing protocol.

Posteroanterior chest DDR was performed using a flat panel detector system (Konica Minolta, Inc., Tokyo, Japan) composed of a flat panel detector (AeroDR fine; Konica Minolta, Inc.) and a pulsed x‐ray generator (RAD speed Pro; Shimadzu Corporation, Kyoto, Japan). Each patient was scanned in the standing position and the pelvis was firmly fastened using a belt (Fig. 2). All patients were instructed regarding the two protocols: breath‐holding protocol and deep‐breathing protocol (Fig. 3). The breath‐holding protocol requested patients to breathe with deep inspiration and hold their breath for 6 s (total time of exposure to radiation: 6 s). The deep‐breathing protocol requested patients to breathe with deep inspiration/expiration for 5 s, and hold their breath for 2 s under the direction of automatic speech (total time of exposure to radiation: 14 s). The exposure conditions were as follows: tube voltage: 95 kV; tube current: 80 mA; duration of pulsed x ray: 4.0 ms; source‐to‐image distance: 1.8 m; and additional filter: 0.2 mm Cu. The additional filter was used to remove soft x rays. The introduction of the Cu filter resulted in a change in the half value layer to 6.58 vs 3.96 mmAl without the Cu filter. The estimated dose per pulse was 0.0074 mGy with a Cu filter and 0.0134 mGy without a Cu filter. The pixel size (400 × 400 µm), overall image area (424.8 × 424.8 mm), gray‐level range of the images 65 536 (16 bits), and signal intensity was proportional to the incident exposure of the x‐ray detector. The dynamic image data, captured at 15 frames/s, were synchronized with the pulsed x ray. As in modern fluoroscopy, chest DDR in this study utilized pulsed x rays. This prevents excessive exposure of the subjects to radiation.

### 2.D Image analysis

**Fig. 4:**
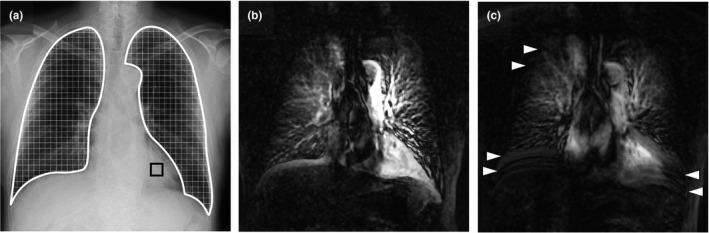
Pulmonary perfusion images and respiratory artifacts in chest digital dynamic radiography (DDR). A 74‐yr‐old male with chronic obstructive pulmonary disease. (a) Original chest radiography. The edges of the lung fields (white frame region) and the region of interest (10 × 10 mm) in the left ventricle (black frame box) were automatically determined. The lung fields were separated using 5‐mm intervals, and changes in the pixel value of each block were analyzed. (b) Maximum intensity projection (MIP) image for chest DDR using the breath‐holding protocol. Excursion of the right diaphragm was 1.6 mm. Entrance surface dose was 1.18 mGy. The mean correlation rates of the bilateral lung fields were 38.7% (right) and 46.6% (left). (c) MIP image for chest DDR using the deep‐breathing protocol. Excursion of the right diaphragm was 2.0 mm. Entrance surface dose was 1.83 mGy. The mean correlation rates of the bilateral lung fields were 30.4% (right) and 35.6% (left). Pulmonary perfusion of the bilateral lower lung fields became vague (blurred image) with the deep‐breathing protocol vs the breath‐holding protocol. Horizontal lines observed in the lung field (white arrowhead) were regarded as incomplete pause of the diaphragm and ribs (misalignment of the rib cage).

During breath‐holding (analyzed phase) in both protocols (Fig. 3), the diaphragm motions and the temporal change in pixel values on sequential chest radiographs data were analyzed using the KINOSIS 1.00 software (Konica Minolta, Inc.). The software was installed in an independent workstation (operating system: Windows 10 professional, Microsoft, Redmond, WA, USA; central processing unit: Intel® Core^TM^ i7‐7500U 2.90 GHz; memory: 8 GB). The edges of the lung fields and the region of interest (ROI) (10 × 10 mm) in the left ventricle were automatically determined. Increases and decreases in pixel values of the left ventricle were determined as the diastole and systole phases, respectively. The lung fields were separated using 5‐mm intervals [Fig. 4(a)]. The cross‐correlation coefficients of the changes of each ROI in the lung fields and left ventricle were calculated. Differences in pixel values between the lung fields and left ventricle were determined throughout one cardiac cycle in each lung field block, representing the difference from the average state in blood volume. From a technical viewpoint, measurements need to be performed in at least three cardiac cycles per image sequence. This is the reason why the breath‐holding protocol was set to 6 s. This image analysis is premised on the synchronization of both ventricles.

Color‐mapping images were produced to visualize the pulmonary perfusion (Videos S1 and S2). This visualizing method is similar to that used by Tanaka et al.,^8^, ^14^ with some modifications. Changes in pixel values were quantified and visualized in the form of a color display (i.e., changes based on a colorimetric scale). Differences in pixel values from baseline were sequentially calculated throughout all frames and superimposed over the original images in the form of a color display. In the color‐mapping images, an increase in blood volume was represented with deep red color. We generated the maximum intensity projection (MIP) image from the color‐mapping images [Figs. 4(b) and 4(c)].

### 2.E Imaging success, correlation rate, and exposure to radiation

In this study, imaging success was defined as right diaphragm excursion of ≤5 mm during breath‐holding (2 s). This is because the size of each ROI in the lung fields was 5 × 5 mm. Diaphragm movement by ≥5 mm could cause respiratory artifacts. We applied the same exposure condition to both protocols. The entrance surface dose was measured as the exposure to radiation, which was calculated assuming a body thickness of 20 cm.

The correlation rate was calculated from the MIP images. We investigated the correlation rate between each pixel values of the lung fields and those of the left cardiac ventricles. The correlation rate calculation involves three steps. First, identification of the pulmonary field and its delimitation into 5‐mm blocks. Second, extraction of the left ventricular signal change during one cardiac cycle. Third, calculation of the percentage of pixels that match the left ventricular signal within these blocks. The lung fields of the MIP were equally divided into three parts from the top (upper lung field, middle lung field, and lower lung field).

### 2.F Lung function tests

All patients underwent lung function tests by routine spirometry using Superior Spiro Discom‐21 FXIII (Chest, Tokyo, Japan). The parameters of forced vital capacity (FVC), percent forced vital capacity (%FVC), forced expiratory volume in one second (FEV_1_), and percent forced expiratory volume in one second (%FEV_1_) were used for the quantitative evaluation of the airway status.

### 2.G Statistical analysis

Qualitative variables are shown as numbers and percentages. Quantitative variables are reported as mean values and standard deviations, unless otherwise indicated. We used the paired t test to compare the imaging success rate, mean excursion of the right diaphragm, exposure to radiation, and the mean correlation rate between the breath‐holding protocol and deep‐breathing protocol. The potential associations between excursion of the right diaphragm and patient characteristics were assessed using Pearson’s correlation coefficient and Student’s *t* test (*R^2^*). All tests were two‐tailed and *P* < 0.05 denoted statistical significance. Data were analyzed with the JMP® 14.0.0 software (SAS Institute Inc., Cary, NC, USA).

## 3 RESULTS

### 3.A Study population

Patient demographics and baseline characteristics are shown in Table 1.

**Table 1:** Patient characteristics.

Age (yr)	68.6 ± 12.3
Sex, n (%)	
Male	32 (76%)
Female	10 (24%)
BMI	22.3 ± 4.3
Heart rate (bpm)	70.9 ± 11.2 bpm
Smoking status, N (%)	
Current or former	32 (76%)
Never	10 (24%)
Respiratory diseases, N (%)	42 (100%)
COPD, N (%)	21 (50%)
Interstitial pneumonia, N (%)	6 (14%)
Nontuberculous mycobacterium, N (%)	6 (14%)
Chronic bronchitis, N (%)	4 (10%)
Acute bronchitis, N (%)	1 (2%)
Bronchial asthma, N (%)	1 (2%)
Pulmonary hypertension, N (%)	1 (2%)
Others (bronchomalacia, malignant lymphoma), N (%)	2 (8%)
Cardiovascular diseases, N (%)	19 (45%)
Hypertension, N (%)	14 (33%)
Stable angina, N (%)	3 (7%)
Aortic regurgitation, N (%)	2 (5%)
Paroxysmal atrial fibrillation, N (%)	2 (5%)
*Peripheral arterial disease, N (%)*	2 (5%)
Old myocardial infarction, N (%)	1 (2%)
Lung function test	
VC (L)	2.99 ± 0.85 L
%VC (%)	96.3 ± 21.0%
FVC (L)	2.94 ± 0.86 L
%FVC (%)	94.9 ± 21.3%
FEV_1_ (L)	1.99 ± 0.66 L
%FEV_1_ (%)	88.0 ± 25.5%
FEV_1_/FVC ratio	68.3 ± 14.3

Unless otherwise indicated, values are presented as the mean ± SD.

BMI: body mass index; COPD: chronic obstructive pulmonary disease; VC: vital capacity; %VC: percent vital capacity; FVC: forced vital capacity; %FVC: percent forced vital capacity; FEV_1_: forced expiratory volume in 1 s; %FEV_1_: percent forced expiratory volume in 1 s; SD: standard deviation.

### 3.B Imaging success rate and exposure to radiation

The imaging success rate was higher with the breath‐holding protocol vs the deep‐breathing protocol (97% vs 69%, respectively) (*P* < 0.0001). The entrance surface dose was lower with the breath‐holding protocol (1.09 ± 0.20 vs 1.81 ± 0.08 mGy, respectively; *p* < 0.0001) (Table 2). In the deep‐breathing protocol, a univariate analysis of associations between excursion of the diaphragm and patient characteristics demonstrated that reduction in FEV_1_ and %FEV_1_ correlated with increase in excursion of the right diaphragm (Table 3). For the breath‐holding protocol, there were no correlations between excursion of the diaphragm and patient characteristics.

**Table 2:** Imaging success rate and exposure to radiation.

	Breath‐holding protocol N = 42	Deep‐breathing protocol N = 42	*P* value
Imaging			
Success, n (%)	41 (97%)	29 (69%)	<0.0001*
Failure, n (%)	1 (2%)	13 (31%)	–
Excursion of the right diaphragm (mm)	1.3 ± 1.2	3.7 ± 3.5	<0.0001*
Exposure to radiation			
Entrance surface dose (mGy)	1.09 ± 0.20	1.81 ± 0.08	<0.0001*

Values are presented as the mean ± SD unless otherwise indicated.

All the results were analyzed using the paired *t* test.

SD, standard deviation.

* *P* < 0.05.

**Table 3:** Univariate analysis of associations between excursion of the diaphragm and patient characteristics.

	Breath‐holding protocol for excursion of the right diaphragm		Deep‐breathing protocol for excursion of the right diaphragm	
	*R* ^2^	*P*‐value**	*R* ^2^	*P* value**
Continuous variables				
Age (yr)	0.001	0.846	0.015	0.436
BMI	0.005	0.660	0.005	0.645
Heart rate	0.009	0.541	0.090	0.053
VC (L)	0.031	0.261	0.024	0.325
%VC (%)	0.010	0.527	0.027	0.297
FVC (L)	0.036	0.225	0.033	0.247
%FVC (%)	0.015	0.441	0.029	0.277
FEV_1_ (L)	0.038	0.218	0.140	0.018*
%FEV_1_ (%)	0.036	0.238	0.111	0.036*
FEV_1_/FVC ratio	0.000	0.932	0.061	0.113

	*R* ^2^	*P*‐value***	*R* ^2^	*P*‐value***
Nominal variables				
Sex	0.001	0.871	0.004	0.698
Cardiovascular diseases	0.030	0.277	0.041	0.210
Smoking status	0.017	0.415	0.079	0.074

BMI: body mass index; VC: vital capacity; %VC: percent vital capacity; FVC: forced vital capacity; %FVC: percent forced vital capacity; FEV_1_: forced expiratory volume in 1 s; %FEV_1_: percent forced expiratory volume in 1 s.

* Indicates *P* < 0.05.

** *P* values were calculated using Pearson’s correlation coefficient.

*** *P* values were calculated using Student’s *t* test.

### 3.C Comparison of the correlation rate between the breath‐holding protocol and deep‐breathing protocol

The correlation rate was higher with the breath‐holding protocol vs the deep‐breathing protocol (Table 4). In the lower lung fields, the correlation rate was markedly different. The difference between the two protocols in the right and left lower lung fields was 15.3% and 14.1%, respectively (both *P* < 0.0001). Compared with each lung field, the correlation rate of the middle lung fields exhibited the highest value in both protocols.

**Table 4:** The correlation rate of temporal changes in each pixel value between the bilateral lung fields and the left cardiac ventricles.

	Breath‐holding protocol N = 42	Deep‐breathing protocol N = 42	*P* value
**Mean correlation rate**			
Right			
Upper lung field (%)	35.5 ± 10.8%	33.3 ± 9.0%	0.065
Middle lung field (%)	46.6 ± 11.0%	39.2 ± 8.1%	<0.0001*
Lower lung field (%)	43.0 ± 7.9%	27.7 ± 5.2%	<0.0001*
Total lung field (%)	41.7 ± 9.3%	33.4 ± 6.6%	<0.0001*
Left			
Upper lung field (%)	38.4 ± 10.6%	37.7 ± 9.1%	0.589
Middle lung field (%)	49.3 ± 9.9%	41.0 ± 8.1%	<0.0001*
Lower lung field (%)	45.0 ± 8.8%	30.9 ± 6.3%	<0.0001*
Total lung field (%)	44.2 ± 8.9%	36.5 ± 7.1%	<0.0001*

Values are presented as the mean ± SD unless otherwise indicated.

All the results were analyzed using the paired *t* test.

SD, standard deviation.

* P < 0.05.

Chest DDR images of a 74‐yr‐old male with chronic obstructive pulmonary disorder (COPD) are presented as an example. Color‐mapping images of chest DDR conducted using both protocols were generated (Videos S1 and S2). The pulmonary perfusion of the bilateral lower lung fields became vague (blurred image) compared with that obtained using the breath‐holding protocol. Horizontal lines observed in the lung field were regarded as incomplete pause of the diaphragm and ribs (misalignment of the rib cage). Similar findings were recorded using the MIP images [Figs. 4(b) and 4(c)].

## 4 DISCUSSION

Digital dynamic radiography is a completely novel modality that can capture images similar to those of pulmonary angiography and scintigraphy^8^, ^11^, ^12^, ^15^, ^16^ in a short time. Moreover, it does not require the use of contrast media. To the best of our knowledge, this is the first report assessing the feasibility of the breath‐holding protocol in patients with various respiratory diseases (mainly COPD). Using DDR for the evaluation of pulmonary perfusion, we identified three strong points of the breath‐holding protocol: high imaging success rate (97%); exposure to low levels of radiation (1.09 ± 0.20 mGy); and significant reduction of respiratory artifacts. For the deep‐breathing protocol, excursion of the diaphragm was related to reduction in FEV_1_ and %FEV_1_. For the breath‐holding protocol, there were no correlations between excursion of the diaphragm and patient characteristics. The reduction of respiratory artifacts noted after using the breath‐holding protocol led to the capture of vivid pulmonary perfusion images, including high concordance with the left cardiac ventricle. In addition to the protocol, there are two other differences between the present study and previous studies.^11^, ^12^, ^15^, ^16^ The first is that the present study included the largest number of patients examined in this field. Thus far, only a few number of case reports, preliminary studies, and animal experiments^14^ have been reported on DDR technology for pulmonary perfusion. The second is that our study clarified the real‐world challenges of the deep‐breathing protocol. Previous studies have shown that the deep‐breathing protocol has the advantage of allowing the simultaneous evaluation of ventilation and blood flow.^8^ However, in some patient groups (e.g., elderly patients or those with respiratory disease), the quality of the pulmonary perfusion images was lower than desired.

Incomplete diaphragm pause could be the main cause of respiratory artifacts. Bizarre diaphragm motion may occur among patients with various respiratory diseases (especially COPD).^17^, ^18^, ^19^ Thus, the imaging protocol is required to reduce excursion of the diaphragm. Yamada *et al*. used chest DDR to investigate excursion of the diaphragm in patients with COPD. They revealed that excursion of the diaphragm was faster and peak diaphragm motion in the inspiratory phase was significantly faster than those observed in healthy subjects.^20^, ^21^ The deep‐breathing protocol requires deep inspiration and expiration in a short time. Hence, it may be difficult for patients to perform this task. For the deep‐breathing protocol in the present study, the reduction in FEV_1_ and %FEV_1_ correlated with an increase in excursion of the right diaphragm (FEV_1_: *R* ^2^ = 0.140, *P* = 0.018; %FEV_1_: *R^2^* = 0.111, *P* = 0.036). These tendencies were in agreement with previous data reported by Yamada *et al*. Greater severity of COPD is associated with the presence of more respiratory artifacts. The breath‐holding protocol is able to assess pulmonary perfusion in all patients, even those with low FEV_1_/%FEV_1_. Furthermore, the shorter examination time resulted in less exposure to ionizing radiation than that linked to the deep‐breathing protocol.

According to the study conducted by Tanaka et al., chest DDR using the deep‐breathing protocol demonstrated an imaging success rate of 71% (10/14 healthy subjects),^12^ and the mean correlation rates were 17–31% among seven healthy subjects.^8^ Those results were similar to the imaging success rate/mean correlation rate of the deep‐breathing protocol in our study (imaging success rate: 69%; mean right correlation rate: 33.4%; mean left correlation rate: 36.5%). The present study did not include participants with pulmonary blood flow abnormality (i.e., pulmonary thromboembolism and pulmonary arteriovenous malformation). Thus, there may not be a large difference in pulmonary blood flow between the population in the studies conducted by Tanaka et al.^8^, ^12^ and that of the present study. Although the deep‐breathing protocol was sufficient to detect pulmonary perfusion in this study, the mean correlation rate of the breath‐holding protocol (right: 41.7%; left: 44.2%) was higher. This result proves that the breath‐holding protocol can stably yield pulmonary perfusion images in concordance with the cardiac cycles.

Respiratory artifacts are categorized into two types according to their appearance: (a) blurred images, which appear as a decrease in the correlation rate in bilateral lower lung fields; and (b) horizontal lines in whole lung fields, which appear as misalignment of the rib cage. In the lower lung fields, the correlation rate was markedly different (right: 15.3% difference; left: 14.1% difference; both lung fields: *P* < 0.0001). This is attributed to reduction of respiratory artifacts by complete diaphragm pause. Generating MIP images from color‐mapping images is helpful for the easy detection of respiratory artifacts [Fig. 4(c)]. Among the three lung fields (upper, middle, and lower), the correlation rate of the middle lung fields tended to be high. A similar result was reported in a previous study conducted by Tanaka et al. on healthy subjects.^8^ This is because the middle lung field includes both main/segmental branches of the pulmonary artery, which have high concordance with the cardiac cycles. In the upper lung field, there were no significant differences between the two protocols, indicating that these areas may not be affected by respiratory artifacts. A physiological study revealed the influence of gravity on the distribution of pulmonary blood flow.^22^ An apical‐to‐basal gradient is established from chest radiography performed in the standing position and posterior–anterior projection. This means that the ability to evaluate perfusion in the lower lung fields is essential. During deep inspiration, pulmonary blood flow increases in parallel with the expansion of all lung lobes.^23^ Thus, our breath‐holding protocol has some advantages, namely the timely detection of pulmonary blood flow, shorter examination time, and exposure to lower levels of radiation.

There are three limitations in our study. First, this was a retrospective study conducted at a single center with a small number of patients, which limits the generalizability of the results. For example, there were only a few females, and patients with interstitial lung disease, bronchial asthma, or lung cancer in this study. Therefore, a larger multicenter study is warranted. Second, many previous studies have used other modalities (contrast CT, magnetic resonance angiography, and scintigraphy) to evaluate pulmonary perfusion. Comparison with other modalities was beyond the scope of this study. Hence, further research to compare chest DDR with other modalities is required. Third, we investigated respiratory artifacts but not body motion artifacts. Color‐mapping/MIP images may also contain body motion artifacts. In this study, we firmly fastened the patients’ lumber with a belt to minimize the body motion artifacts.

## 5 CONCLUSION

We have illustrated a new approach to image pulmonary perfusion by improving the protocol of chest DDR. For the evaluation of pulmonary perfusion, the breath‐holding protocol was more beneficial than the deep‐breathing protocol among patients with respiratory diseases, and resulted in a significant reduction of respiratory artifacts. Vivid images of pulmonary perfusion can be obtained through a simple and rapid examination. We are confident that our research will serve as a basis for future studies on various abnormalities of pulmonary perfusion.

## AUTHORS’ CONTRIBUTION

GT, YK, YI, and FS recruited the participants of this study. SY wrote the primary draft of this manuscript, and TH is a mentor to SY. FS and TH equally contributed to this work. KT, SK, TM, and YI reviewed the final manuscript. All authors read and approved the final manuscript.

## CONFLICT OF INTEREST

No conflict of interest.

## ETHICAL GUIDELINES

This study has been reviewed based on the Clinical Trials Act and approved as a specified clinical trial, entitled “Development and evaluation of chest functional examination technology with flat panel detector (jRCTs052180103).”

## Supporting information


**Video S1**. Pulmonary perfusion video for chest digital dynamic radiography (DDR) using the breath‐holding protocol.

**Video S2**. Pulmonary perfusion video for chest digital dynamic radiography (DDR) using the deep‐breathing protocol.
